# Endocan as a prognostic biomarker of triple-negative breast cancer

**DOI:** 10.1007/s10549-016-4057-8

**Published:** 2016-11-25

**Authors:** Atsunobu Sagara, Katsuhide Igarashi, Maky Otsuka, Akihiro Kodama, Mutsumi Yamashita, Rei Sugiura, Takeshi Karasawa, Kazuhiko Arakawa, Michiko Narita, Naoko Kuzumaki, Minoru Narita, Yoshinori Kato

**Affiliations:** 1 Department of Pharmacology, Hoshi University School of Pharmacy and Pharmaceutical Sciences, 2-4-41 Ebara, Shinagawa-ku, Tokyo, Japan; 2 Life Science Tokyo Advanced Research Center (L-StaR), Hoshi University School of Pharmacy and Pharmaceutical Sciences, 2-4-41 Ebara, Shinagawa-ku, Tokyo, Japan

**Keywords:** Triple-negative breast cancer, Endocan, ESM-1, Prognostic biomarker, MDA-MB-231, MDA-MB-231BR

## Abstract

**Purpose:**

Triple-negative breast cancer (TNBC) has aggressive characteristics and fewer treatment options than other subtypes. The purpose of this study was to explore prognostic biomarkers for TNBC that can be easily detected from the blood samples.

**Methods:**

MDA-MB-231 and MDA-MB-231BR, a brain metastatic variant of the human TNBC cell line MDA-MB-231, were used as less and more aggressive models of TNBC, respectively. The extent to which the candidate gene/protein identified by RNA sequencing correlated well with aggressiveness of TNBC and how much protein was detected from the blood of tumor-bearing mice were evaluated.

**Results:**

Both the in vitro proliferation and in vivo tumor growth of MDA-MB-231BR were more rapid than those of MDA-MB-231. RNA sequencing identified * ESM1* as a gene that was expressed significantly more in MDA-MB-231BR than in MDA-MB-231, and qRT-PCR confirmed a significantly higher expression of * ESM1* in MDA-MB-231BR xenograft in vivo. Furthermore, Kaplan–Meier analysis of relapse-free survival demonstrated that TNBC patients with high * ESM1* expression had clearly worse relapse-free survival than those with low ESM1 expression, which was consistent with our preclinical findings. Endocan, a protein of * ESM1* gene product, was successfully detected in both conditioned medium from MDA-MB-231BR and plasma samples from mice bearing MDA-MB-231BR xenograft, which showed a significantly distinct pattern from less aggressive MDA-MB-231. Moreover, bisulfite sequence analysis revealed that overexpression of* ESM1* in MDA-MB-231BR might be attributed to DNA demethylation in an upstream region of the* ESM1* gene.

**Conclusion:**

This study indicates that endocan could be used as a blood-based prognostic biomarker in TNBC patients.

**Electronic supplementary material:**

The online version of this article (doi:10.1007/s10549-016-4057-8) contains supplementary material, which is available to authorized users.

## Introduction

Triple-negative breast cancer (TNBC) poses a serious problem in women who are, in particular, young [1], African-American, or have BRCA1 gene mutations [2–4], due to its aggressive characteristics as well as lack of targeted therapy. Although human epidermal growth factor receptor type 2 (HER2)-positive breast cancers have a poor prognosis [5], the development of therapeutic monoclonal antibodies against HER2 receptor has drastically improved the prognosis of such breast cancers. Unfortunately, no FDA-approved drugs are currently available to substantially improve the survival of TNBC patients compared to classical anticancer agents. TNBC patients, however, do not always have a poor prognosis and, thus, predicting the prognosis of TNBC patients prior to treatment would be of value in assessing the future condition of TNBC patients and their quality of life. Biopsy of the tumor and subsequent histological analysis can help to determine the subtype of breast cancer, the therapeutic regimen, and possibly the prognosis [6, 7]. It would be desirable to foresee the prognosis of TNBC patients through the use of a more patient-friendly technique, such as blood withdrawal.

In this study, we used the human TNBC cell line MDA-MB-231, an often-used model of human TNBC cells, and its brain metastatic phenotype MDA-MB-231BR. MDA-MB-231BR was originally selected from MDA-MB-231 that had repeatedly metastasized to the brain following the injection of the cells into the left ventricle of mice, and MDA-MB-231BR has been reported to show a higher degree of malignancy than its parent cell line MDA-MB-231 [8, 9]. Therefore, we used MDA-MB-231 and MDA-MB-231BR as less and more aggressive models of TNBC, respectively. In the current study, MDA-MB-231BR indeed showed a significantly higher proliferation rate in vitro and a significantly faster growth rate in vivo and, thus, we explored the genetic difference between MDA-MB-231 and MDA-MB-231BR. We evaluated the correlation between the prognosis of TNBC and gene expression, and quantified the candidate protein, endocan, excreted from the cells in vitro and in the blood of mice bearing orthotopic MDA-MB-231 or MDA-MB-231BR xenograft to assess the potential for endocan to serve as a blood-based prognostic biomarker of TNBC.

## Materials and methods

### Reagents

Medetomidine hydrochloride (MED), midazolam (MID), and butorphanol tartrate (BUT) were purchased from Nippon Zenyaku Kogyo Co., Ltd. (Fukushima, Japan), Sandoz (Holzkirchen, Germany), and Meiji Seika Pharma Co., Ltd. (Tokyo, Japan), respectively. The mixture of MED, MID, and BUT was used as an anesthetic, and injected at doses of 0.3, 4, and 5 mg/kg, respectively [10]. ECM gel was obtained from Sigma-Aldrich Co. (St. Louis, MO). A human endocan ELISA kit (EndoMark^®^ H1) was purchased from Lunginnov SAS (Lille, France).

### Cell culture

The human breast carcinoma cell line MDA-MB-231 (ECACC, Salisbury, UK), its brain metastatic variant MDA-MB-231BR, bone metastatic variant MDA-MB-231SCP2, and murine TNBC cell line 4T1/luc2 (PerkinElmer, Waltham, MA) were cultured in RPMI-1640 (Thermo Fisher Scientific Inc., Waltham, MA) with 10% fetal bovine serum. MDA-MB-231BR and MDA-MB-231SCP2 were kind gifts from Dr. Patricia Steeg and Dr. Joan Massagué, respectively. Contamination by *Mycoplasma* or fungi was routinely checked, and the cells were treated or discarded when necessary; only uncontaminated cells were used. All cells were maintained under a humidified atmosphere of 5% CO_2_ at 37 °C.

### Cell proliferation assay

The proliferation rate for each cell line was determined using a Cell Counting Kit-8 (CCK-8; Dojindo Laboratories, Kumamoto, Japan). Cells (1 × 10^3^ or 2 × 10^3^ cells/well) were cultured in a 96-well plate, and the number of viable cells was determined at 0, 24, 48, and 72 h using a microplate reader (at 450 nm) after 4 h of incubation with a CCK-8 reagent. The doubling time was calculated as ${\text{doubling time}} = 48 \times \frac{\log 2}{{\log \left( {\# {\text{of cells at}} 72 {\text{h}}} \right) - \log (\# {\text{of cells at }}24 {\text{h}})}}.$The cell proliferation experiments were independently repeated at least four times.

### Animals

Female athymic *nu*/*nu* mice (Balb/c background, 4 weeks of age, 17–20 g) were purchased from CLEA Japan, Inc. (Tokyo, Japan).

### Tumorigenesis in mammary fat pad

Mammary MDA-MB-231 or MDA-MB-231BR tumor models were established by inoculating 1 × 10^6^ cells dispersed in 50 μL of 50% ECM gel/Hanks’ balanced salt solution into the third mammary fat pad of the mice. The size of xenografted tumors was measured once or twice a week in a blinded manner using a caliper. The tumor volume was calculated as ${\text{Tumor volume}} \left( {{\text{mm}}^{ 3} } \right) = \frac{{\left( {\text{length}} \right) \times \left( {\text{width}} \right)^{2} \times \pi }}{6} .$

### RNA sequencing

Total RNA was isolated from each cell line using a mirVana™ Isolation Kit (Thermo Fisher Scientific Inc.) following the manufacturer’s instructions. Libraries for RNA-seq were constructed using SMARTer Stranded Total RNA-Seq Kit-Pico Input Mammalian (Takara Bio, Shiga, Japan) according to the manufacturer’s instructions. All libraries were sequenced on MiSeq (Illumina, Inc., San Diego, CA) with paired-end read (75 bp). The 75-bp-long paired-end sequence reads were mapped to a human genome reference sequence (hg19) using CLC Genomics Workbench software (CLC bio, Aarhus, Denmark), and the mapped data were exported as BAM files and imported to Strand NGS analysis software (Agilent Technologies, Santa Clara, CA) and used for downstream gene expression analysis.

### qRT-PCR

Total RNA was isolated from each cell line or xenograft tumors using RNeasy (QIAGEN, Hilden, Germany), according to the manufacturer’s instructions. Reverse-transcription was performed with the PrimeScript RT Master Mix (Takara Bio Inc.). qPCR was performed using SYBR Premix Ex Taq II (Takara Bio Inc.), and an Applied Biosystems^®^ StepOnePlus™ Real-Time PCR System (Thermo Fisher Scientific Inc.). The PCR primer sets used are shown in Supplementary Table S1. The thermal cycle profile was as follows: (1) activation at 95 °C for 30 s; (2) denaturation at 95 °C for 5 s; and (3) annealing and extension at 60 °C for 30 s. PCR amplification was performed for 40 cycles. Data are expressed as the expression relative to RPS18 mRNA as a housekeeping gene using the $2^{{ - \Delta \Delta C_{\text{T}} }}$ method.

### siRNA delivery

SMARTpool: Accell Human *ESM1* siRNA (GE Healthcare Dharmacon Inc., Lafayette, CO) or control siRNA were transfected into MDA-MB-231BR at a final concentration of 1 µM with Accell siRNA delivery media with 2.5% FBS. Complexes of siRNA were added to MDA-MB-231BR cultured in 24-well and 96-well plates, and *ESM1* gene expression and proliferation rates were assessed. The knockdown efficiency and doubling time were determined by a quantitative RT-PCR and a CCK-8 assay, respectively, both of which were carried out 72 h after transfection. At least three independent samples were collected and used for each experiment.

### Enzyme-linked immunosorbent assay (ELISA) for the quantification of endocan

To quantify endocan secreted from the cells, 1 × 10^5^ cells were cultured on 6-well plates. The conditioned media were collected after 72 h of incubation and stored at −20 °C until use. The number of live cells in each well was counted using the trypan blue exclusion assay. To quantify endocan circulating in the bloodstream, blood was collected from mice bearing MDA-MB-231 or MDA-MB-231BR, and the plasma was isolated and stored at −80 °C until use. Endocan was quantified by sandwich ELISA. Conditioned media were collected from five independent samples of each cell line (*N* = 5), and plasma was collected from seven mice from each xenograft model (*N* = 7).

### Kaplan–Meier analysis of relapse-free survival in different molecular subtypes of breast cancer patients

Kaplan–Meier curves of relapse-free survival were generated and analyzed using the online resource http://kmplot.com/analysis [11]. The conditions used to generate Kaplan–Meier curves were as follows: Affymetrix ID = 208394 × at (*ESM1*); Survival = RFS; Split patients by = median; and Follow-up threshold = all. To classify molecular subtypes, receptor statuses were set at ER = negative, PR = negative, and HER2 = negative for TNBC patients, and classifications for other subtypes (luminal A, luminal B, and HER2-positive) were selected from a pull-down menu. For other conditions, such as lymph node status, grade, TP53 status, “all” was selected for all molecular subtypes. A 2014 version of the database was used.

### Bisulfite sequencing analysis

One microgram of genomic DNA was used for sodium bisulfite treatment using the MethylEasy Xceed Rapid DNA Bisulphite Modification Kit (Takara Bio Inc.) according to the manufacturer’s instructions. Several regions in the *ESM1* genomic region around the transcription start site were PCR-amplified from bisulfite-treated genomic DNA. The following primers were used: Region1: *ESM1*-1-FW (5′-TTGGTTTTAGTTGTTATTTGGAG-3′) and *ESM1*-1-RV (5′- CCCTCAACACTATAACAACAATAA-3′). Region2: *ESM1*-2-FW (5′-ATTGTTGTTATAGTGTTGAGGG-3′) and *ESM1*-2-RV (5′-AATTTTTCAATCCTCATACAAA-3′). Region3: *ESM1*-3-FW (5′-AAATTGGTAGTTGAGTTTTTGTTTA-3′) and *ESM1*-3-RV (5′-CTCTAAAACAAAACTACACCTCAA-3′). The PCR products were cloned into the pCR4-TOPO vector (Thermo Fisher Scientific Inc.), and clones were randomly selected and amplified for sequencing with an illustra TempliPhi DNA Amplification Kit (GE Healthcare, Little Chalfont, UK) according to the manufacturer’s instructions. The proportion of methylated cytosine was calculated after sequencing at least 12 clones.

### Statistical analysis

The normality of the data distribution was estimated using StatPlus^®^: mac software (AnalystSoft Inc., Alexandria, VA, USA). The statistical difference between two means was determined by a two-tailed unpaired *t* test, and the statistical difference between two medians was determined by a Mann–Whitney U test. For comparison among multiple means, one-way Analysis of Variance (ANOVA) was used for omnibus *F*-test, and at least one contrast among the means was considered significant when the *F* value was greater than the critical value of the *F*-distribution. The Tukey’s test was used for post hoc analysis. The plasma endocan levels in MDA-MB-231- and MDA-MB-231BR-bearing mice were compared using a nonparametric Kolmogorov–Smirnov test. Differences between the groups were considered significant if the *P*-value was less than 0.05.

## Results

### MDA-MB-231BR was more malignant than MDA-MB-231

**Fig. 1:**
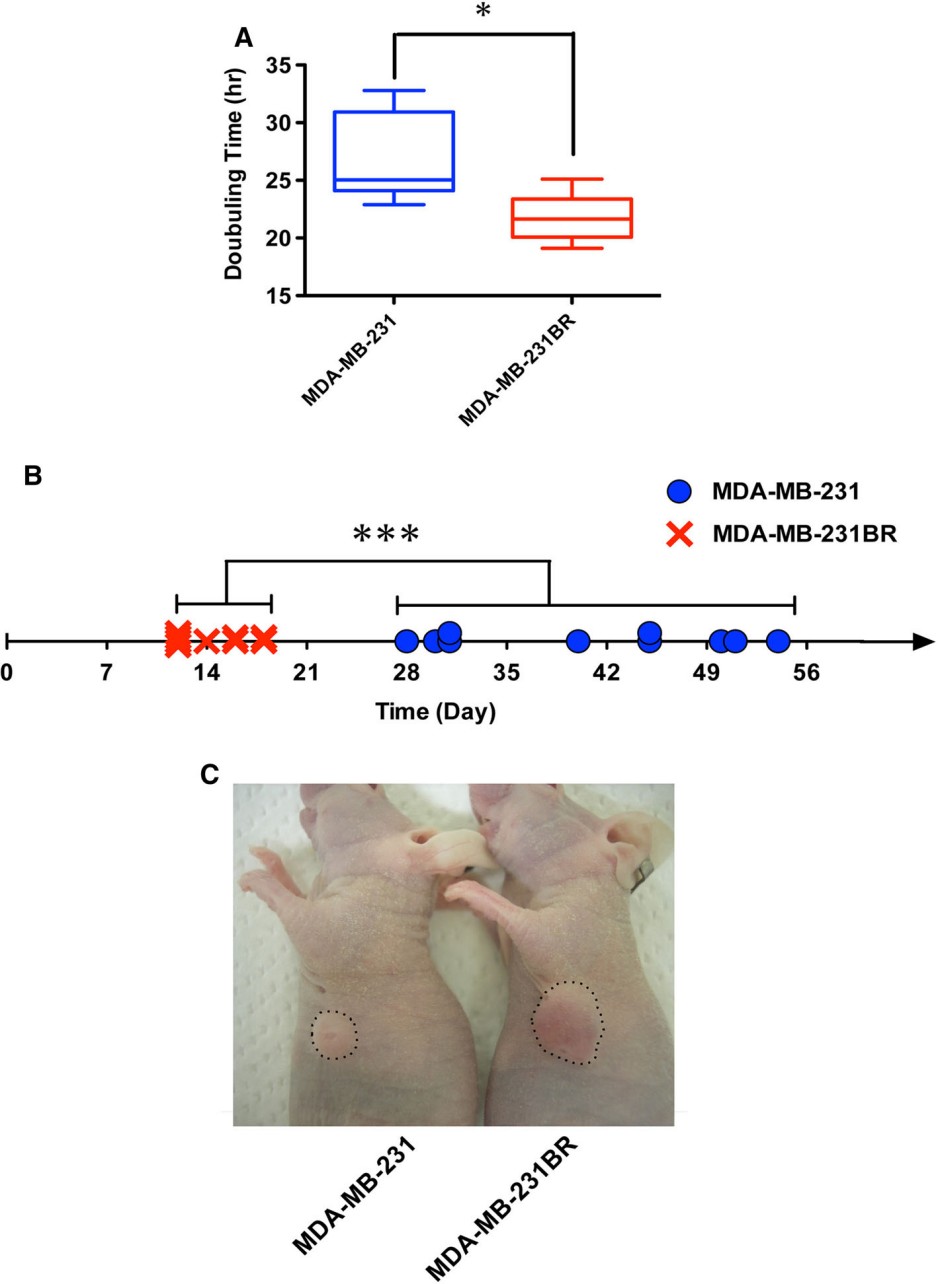
In vitro proliferation and in vivo tumor growth of MDA-MB-231 and MDA-MB-231BR. **a** Doubling time of MDA-MB-231 and MDA-MB-231BR grown in RPMI-1640 with 10% FBS (*N* = 6). The band within the *box* represents the median, and the *upper* and *lower whiskers* represent the maximum and minimum values, respectively. * *P* < 0.05. **b** Days at which the tumors reached a volume of 100 mm^3^ in individual mice. *** *P* < 0.001. **c** Representative mice bearing MDA-MB-231 and MDA-MB-231BR on Day 19

Cancer cells with rapid proliferation, drug resistance, and metastatic potential are considered to be more malignant, and we sought to compare in vitro proliferation and in vivo tumor growth of MDA-MB-231 and MDA-MB-231BR. As shown in Fig. 1a, the doubling time of MDA-MB-231BR (21.6 h) was significantly shorter than that of MDA-MB-231 (25.1 h), indicating that MDA-MB-231BR showed more rapid proliferation. Similarly, the MDA-MB-231BR mammary xenograft reached a volume of 100 mm^3^ at between 12 and 18 days, which was significantly faster than the growth of MDA-MB-231 (between 28 and 54 days) (Fig. 1b). A representative image of mice bearing MDA-MB-231BR and MDA-MB-231 is shown in Fig. 1c. Based on these results, MDA-MB-231BR and MDA-MB-231 were used as more and less aggressive models of TNBC, respectively, in this study.

### MDA-MB-231BR significantly overexpressed *ESM1* compared to the expression in MDA-MB-231 in vitro and in vivo

**Fig. 2:**
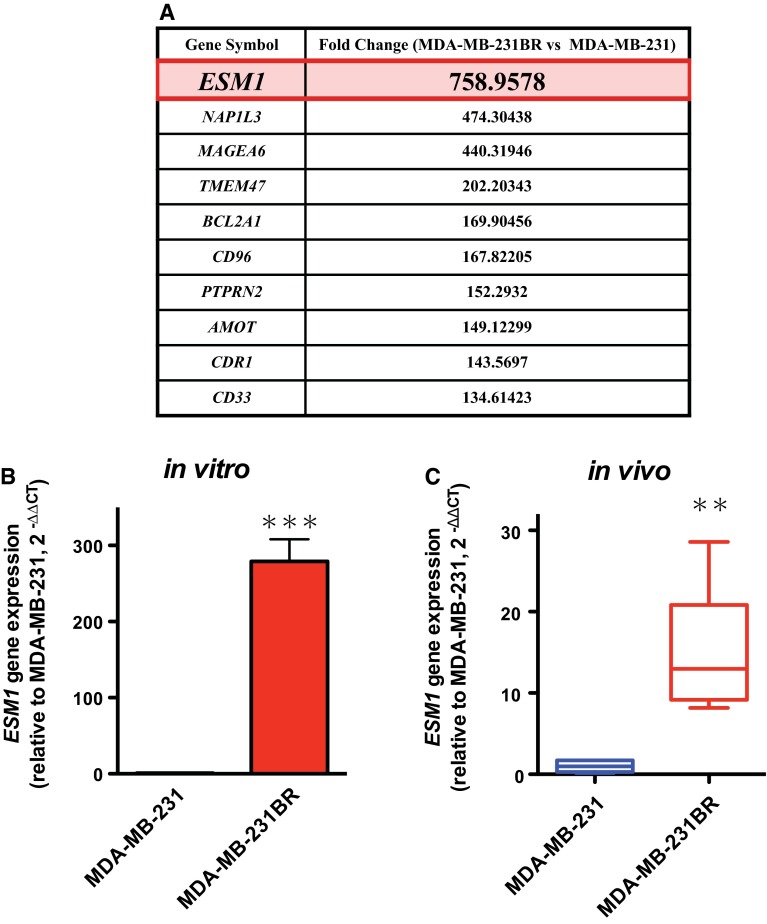
*ESM1* expression levels in MDA-MB-231 and MDA-MB-231BR. **a** Top 10 upregulated genes in MDA-MB-231BR compared to MDA-MB-231. **b** *ESM1* expression levels in MDA-MB-231 and MDA-MB-231BR in vitro. Data represent the mean with SEM of at least seven independent samples (*N* = 7 for MDA-MB-231; *N* = 8 for MDA-MB-231BR). *** *P* < 0.001. **c** *ESM1* expression levels in MDA-MB-231 and MDA-MB-231BR xenografts. The band within the *box* represents the median, and the *upper* and *lower whiskers* represent the maximum and minimum values, respectively. ** *P* < 0.01

RNA sequencing revealed that *ESM1* was the gene that was most upregulated in MDA-MB-231BR relative to the expression in MDA-MB-231 (Fig. 2a). The elevated expression of *ESM1* in MDA-MB-231BR compared to MDA-MB-231 was validated by quantitative RT-PCR (Fig. 2b). The overexpression of *ESM1* was also observed in MDA-MB-231BR xenograft grown in mammary fat pad (Fig. 2c).

**Fig. 3:**
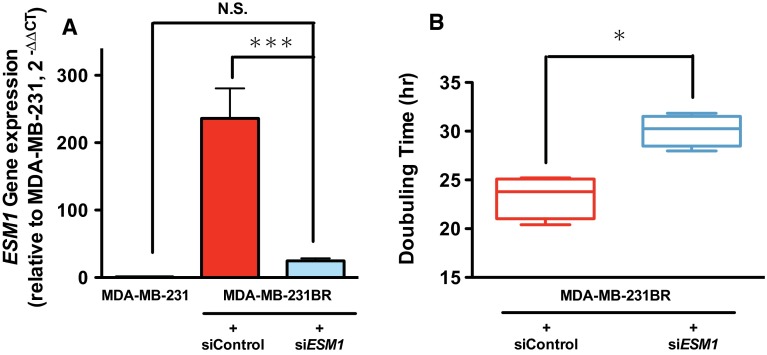
Effect of specific knockdown of *ESM1* on MDA-MB-231BR. **a** Changes in gene expressions of *ESM1* in MDA-MB-231BR in response to *ESM1* siRNA. Gene expression levels were expressed relative to the value in MDA-MB-231 calculated by the $2^{{ - \Delta \Delta C_{\text{T}} }}$ method. Data represent the mean with SEM of three independent samples (*N.S.* No significance; *** *P* < 0.001). **b** Doubling time of MDA-MB-231BR treated with control siRNA and *ESM1* siRNA when grown in RPMI-1640 with 10% FBS (*N* = 4). The band within the *box* represents the median, and the *upper* and *lower whiskers* represent the maximum and minimum values, respectively. * *P* < 0.05

To investigate the extent to which upregulated *ESM1* contributed to rapid growth of MDA-MB-231BR, we knocked down *ESM1* gene in MDA-MB-231BR with siRNA, and assessed its proliferation rate. Human *ESM1* siRNA successfully downregulated *ESM1* in MDA-MB-231 up to the level in MDA-MB-231, which resulted in the prolongation of doubling time of MDA-MB-231 (Fig. 3).

### *ESM1* expression could reflect the prognosis of TNBC patients

**Fig. 4:**
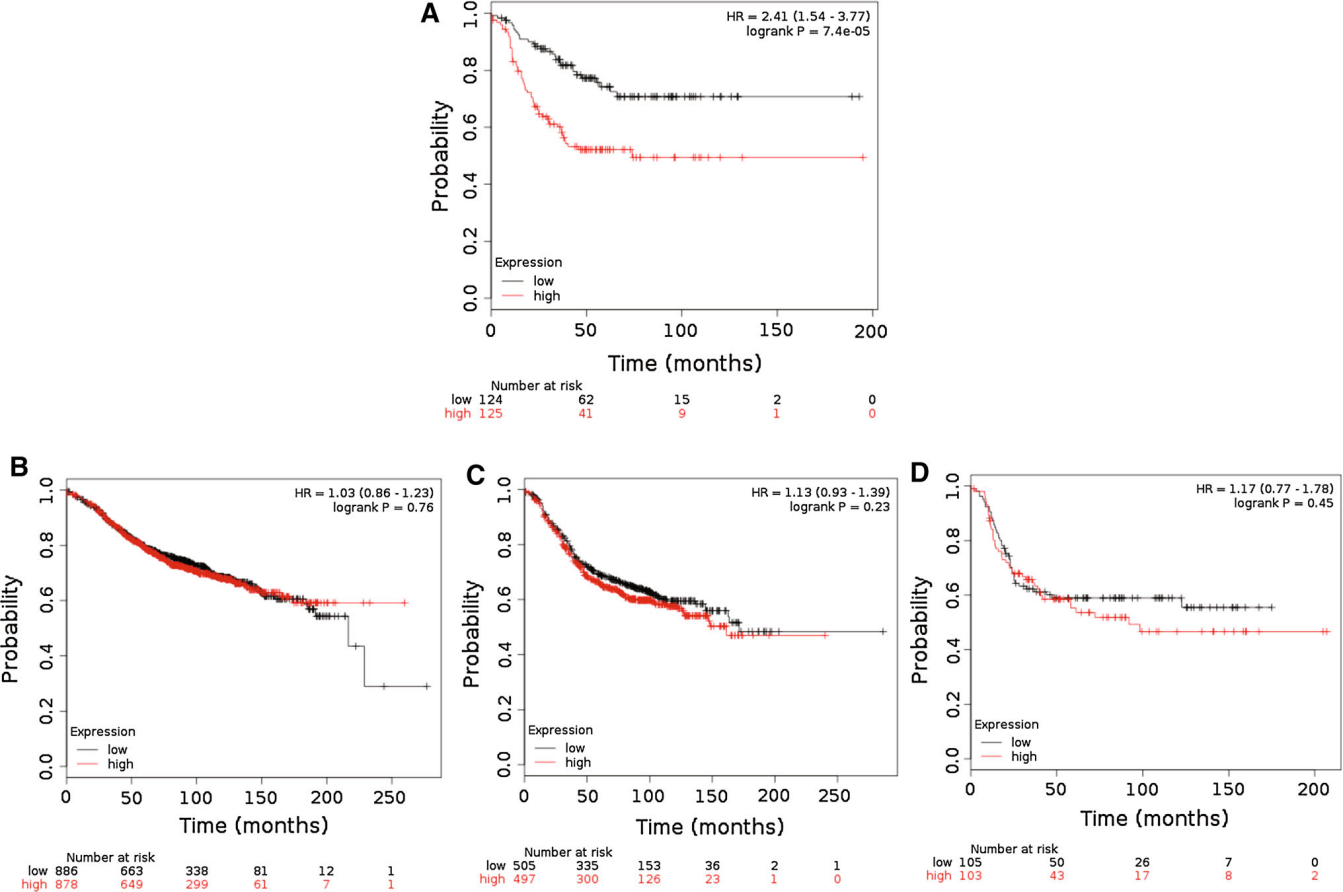
Kaplan–Meier plots of relapse-free survival based on *ESM1* expression in patients with four different subtypes of breast cancer. **a** TNBC. **b** Luminal A. **c** Luminal B. **d** HER2

To support our preclinical data, the relapse-free survival of breast cancer patients with either high or low expression of endocan was determined using an online tool for each subtype of breast cancer. The Kaplan–Meier plotter provided unequivocal evidence that TNBC patients with high *ESM1* expression had a worse relapse-free survival than those with low *ESM1* expression (Fig. 4a). Interestingly, the expression of *ESM1* did not have any impact on the prognosis of other subtypes of breast cancer (Figs. 4b–d). The other genes listed in Fig. 2a had little influence on the prognosis of TNBC patients (Supplementary Fig. S1). Accordingly, *ESM1* expression could be a good indicator of the prognosis of TNBC patients.

### Extracellular protein levels of endocan excreted from MDA-MB-231BR were significantly higher than those from MDA-MB-231 in vitro and in vivo

**Fig. 5:**
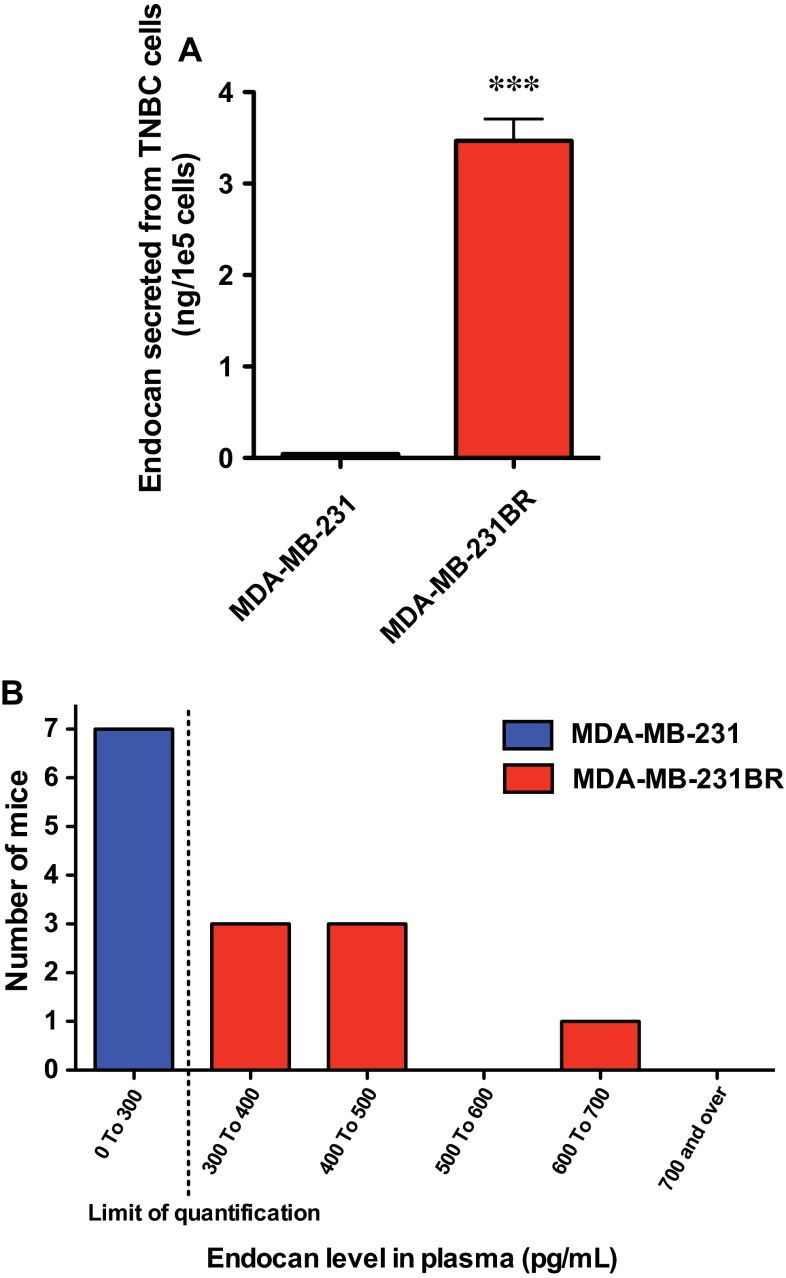
Extracellular endocan levels in MDA-MB-231 and MDA-MB-231BR. **a** Conditioned media from MDA-MB-231 and MDA-MB-231BR. Data represent the mean with SEM of five independent samples. *** *P* < 0.001. **b** Histogram of endocan levels in plasma obtained from mice bearing MDA-MB-231 and MDA-MB-231BR (*N* = 7 for each group). The plasma endocan level in MDA-MB-231-bearing mice was below the limit of quantification. The two-sample Kolmogorov–Smirnov test confirmed that the plasma endocan levels in MDA-MB-231-and MDA-MB-231BR-bearing mice were significantly different (*P* < 0.001)

To investigate endocan secreted from cells into the extracellular space, we examined endocan protein in the supernatant of cell culture using ELISA. Consistent with the results of qRT-PCR in these cell lines, the extracellular protein level of endocan in MDA-MB-231BR was significantly elevated relative to that in MDA-MB-231 (Fig. 5a). Moreover, to investigate the usefulness of endocan as a blood-based biomarker, we checked its plasma levels in MDA-MB-231 and MDA-MB-231BR xenograft models. The endocan protein was detected only from the plasma of the MDA-MB-231BR-bearing mice, but not from the MDA-MB-231-bearing mice (Fig. 5b). Taken together, these results indicate that endocan could be used as a blood-based prognostic biomarker for TNBC.

### DNA demethylation of *ESM1* promoter coincides with the upregulation of *ESM1* in MDA-MB-231BR

**Fig. 6:**
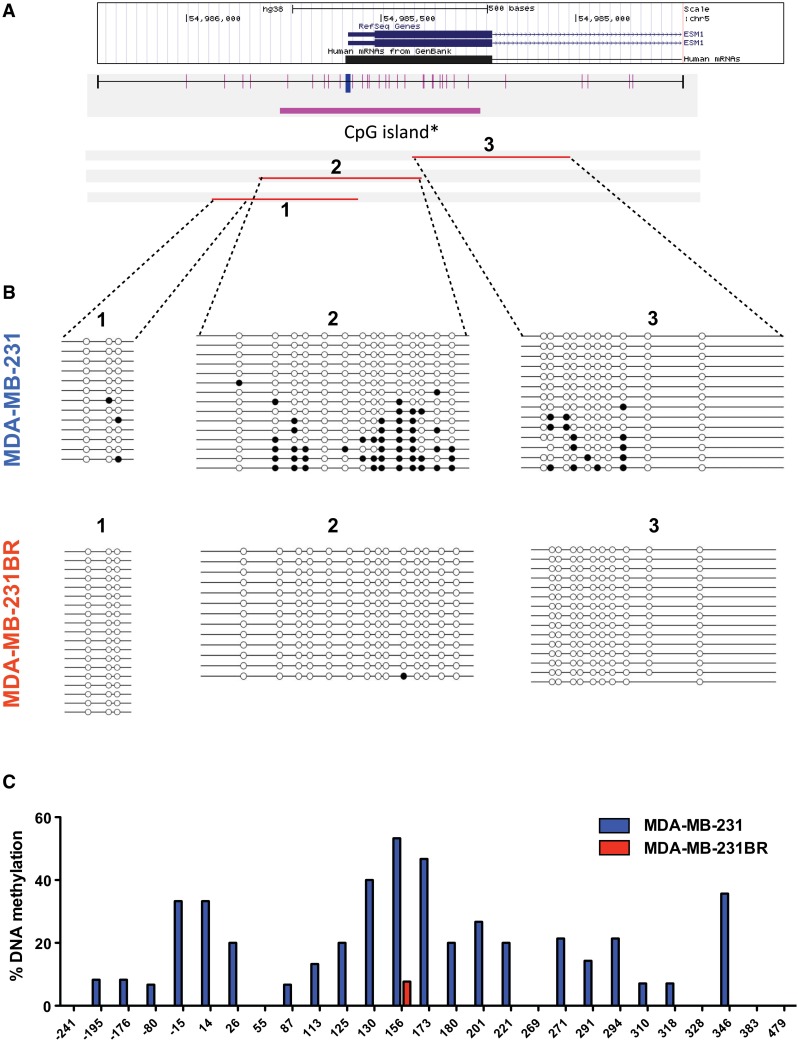
DNA methylation status of CpG islands upstream of the *ESM1* gene determined by sequencing after bisulfite modification of genomic DNA. **a** Locations of CpG sites upstream of the *ESM1* gene and bisulfite sequencing (BS) primers used in this study. **b** Methylation status of CpG sites in MDA-MB-231 and MDA-MB-231BR. **c** Percent DNA methylation in regions designated in (**a**)

We next compared the methylation status of *ESM1* promoter in MDA-MB-231 and MDA-MB-231BR. DNA methylation occurred in at least 20% of MDA-MB-231’s population which accounted for about half of the CpG sites, whereas the CpG sites of *ESM1* promoter in MDA-MB-231BR were almost completely demethylated (Fig. 6). Figure 6 also shows that the population of MDA-MB-231 was heterogenous. Taken together, these results show that epigenetic changes had a significant impact on the characteristics of MDA-MB-231BR, and DNA demethylation at the *ESM1* promoter could be part of the reason why MDA-MB-231BR overexpresses *ESM1*.

## Discussion

MDA-MB-231BR has previously been reported to show greater metastatic potential [8, 12, 13] and drug resistance than MDA-MB-231 [9]. In this study, both in vitro and in vivo studies demonstrated that MDA-MB-231BR was more malignant than MDA-MB-231. MDA-MB-231BR xenograft in mammary fat pad constantly reached a volume of 100 mm^3^ at around two weeks after inoculation, whereas a few MDA-MB-231 xenografts failed to develop tumors. It has been identified that MDA-MB-231 consists of a heterogeneous population of TNBC cells [14], while MDA-MB-231BR that we used here is likely to be a more selected population of MDA-MB-231 because of its brain metastatic variant. This partly explains the heterogeneous tumor growth of MDA-MB-231 and the constantly rapid growth of MDA-MB-231BR. Despite a metastatic phenotype, the proliferation rate of MDA-MB-231SCP2 was comparable to that of MDA-MB-231 (Supplementary Table S2a).

RNA sequencing is a powerful tool for analyzing the transcriptome in cells, and permits comparisons of the genetic backgrounds of multiple cell lines [15]. In this study, we found that *ESM1* expression in MDA-MB-231BR was significantly higher than that in MDA-MB-231. Endocan was originally identified as an endothelial cell-specific molecule-1 (ESM-1), a soluble dermatan sulfate proteoglycan that was secreted from endothelial cells [16]. Later, increasing experimental evidence revealed that endocan levels were increased in sepsis [17, 18], obesity [19], and some types of cancer [20–22]. Endocan has also reportedly been found in cancer cells [23, 24], and intracellular human endocan had a functional role in regulating cell growth and facilitating tumor growth [25]. This is likely to hold true for human TNBC cell lines; *ESM1*-overexpressing human TNBC cell lines demonstrate a high proliferation rate compared to human TNBC cell lines with low *ESM1* expression (Fig. 1; Supplementary Table S2a), which was not the case with mouse TNBC cell line 4T1/luc2 (Supplementary Table S2b). In fact, mouse endocan is less glycanated than human endocan, and its biological properties are completely opposite to human endocan [26, 27]. Unlike MDA-MB-231SCP2, some other metastatic human TNBC cell lines were reported to overexpress *ESM1* [28]; however, no proliferation rates were available for those cell lines. These observations may indicate that overexpression of human *ESM1* leads to rapid proliferation and might result in distant metastases for human TNBC cells, but also imply that metastatic human TNBC cells might not necessarily overexpress human *ESM1*. Based on our results and earlier reports on human *ESM1*/endocan [29–31], the overexpression of human *ESM1* could be part of the reason why MDA-MB-231BR was more malignant than MDA-MB-231.

The Kaplan–Meier plotter is a useful online tool for exploring biomarkers for several cancers, including breast cancers [11], and has been used in many studies. A high expression of *ESM1* permitted the identification of TNBC patients with a poor outcome, whereas there were no correlations between the expression of *ESM1* and the prognosis of patients with other subtypes of breast cancer. A high endocan level has been reported to show a good correlation with a poor prognosis and metastasis in several types of cancer [32–34]. Endocan circulating in the bloodstream has also been reported to be increased with increasing tumor volume [25]. With respect to breast cancers, endocan could be a useful indicator for predicting the outcome of TNBC patients, but not the outcome of patients with other subtypes of breast cancer. Although other factors cannot be ruled out, our findings were consistent with the report that endocan is a key factor in accelerated tumor growth in TNBC [21]. Further studies will be needed to unravel the mechanism by which endocan leads to a poor outcome specifically in TNBC patients.

DNA methylation suppresses gene transcription, while DNA demethylation promotes gene transcription [35]. To investigate whether DNA demethylation at the *ESM1* promoter in MDA-MB-231BR affected the upregulation of *ESM1* and subsequent endocan production, we treated each cell line with the bisulfite assay. Another key finding of the present study was complete DNA demethylation at the *ESM1* promoter, which was observed with the upregulation of *ESM1* and increased subsequent endocan secretion from MDA-MB-231BR. However, it is not yet clear what triggers epigenetic changes at the *ESM1* promoter in breast cancer cells.

In conclusion, endocan was constantly detected in the plasma of mice bearing MDA-MB-231BR, an aggressive model of TNBC, even in tumors with a volume of less than 100 mm^3^. Epigenetic modification might lead to this event. The Kaplan–Meier plot supports our observation of a correlation between endocan/ESM1 levels and the aggressiveness of TNBC cell lines. These results indicate that the prognosis of TNBC patients could easily be predicted by measuring plasma endocan levels through simple blood withdrawal. Further clinical studies will be needed to ensure the clinical utility of this strategy.

## Electronic supplementary material

Below is the link to the electronic supplementary material.

Supplementary material 1 (DOCX 64 kb)

Supplementary material 2 (DOCX 215 kb)
