# Supplementary material for: Endocan as a prognostic biomarker of triple-negative breast cancer
Source: Breast Cancer Res Treat. 2016 Nov 25;161(2):269–78. doi: 10.1007/s10549-016-4057-8 (PMC5225208; doi:10.1007/s10549-016-4057-8)
Supplement: Supplementary file 1 — Supplementary material 1 (DOCX 64 kb) [file 10549_2016_4057_MOESM1_ESM.docx]

**Endocan as a prognostic biomarker of triple-negative breast cancer**

Atsunobu Sagara^1^ ∙ Katsuhide Igarashi^2^ ∙ Maky Otsuka^2^ ∙ Akihiro Kodama^1^ ∙ Mutsumi Yamashita^1^ ∙ Rei Sugiura^1^ ∙ Takeshi Karasawa^1^ ∙ Kazuhiko Arakawa^1^ ∙ Michiko Narita^1^ ∙ Naoko Kuzumaki^1^ ∙ Minoru Narita^1,2^ ∙ Yoshinori Kato^2^

^1^ Department of Pharmacology, Hoshi University School of Pharmacy and Pharmaceutical Sciences, 2-4-41 Ebara, Shinagawa-ku, Tokyo, Japan

^2^ Life Science Tokyo Advanced Research Center (L-StaR), Hoshi University School of Pharmacy and Pharmaceutical Sciences, 2-4-41 Ebara, Shinagawa-ku, Tokyo, Japan

Corresponding author:

* Yoshinori Kato, Phone: +81-3-5498-5844; E-mail: y-kato@hoshi.ac.jp

* Minoru Narita, Phone: +81-3-5498-5784; E-mail: narita@hoshi.ac.jp

# of Words (Abstract): 248

# of Words (Text): 3,340

# of Figures/Tables: 6

# of Supplementary Figures/Tables: 3

**Supplementary Method**

**Transduction of a lentiviral vector carrying an *ESM1* gene into MDA-MB-231**

To generate stably-transduced clone of MDA-MB-231/ESM1, MDA-MB-231 was seeded in a 24-well plate, and transduced with human *ESM1*-expressing lentiviral packaging particles (Origene Technologies, Inc., Rockville, MD) in 500 µL RPMI-1640 with 10% fetal bovine serum in the presence of polybrene (Sigma-Aldrich) with a final concentration of 8 µg/mL. After four-hour incubation, transduced cells were washed with fresh medium, and continued to be cultured as per normal. Several colonies were subcultured each passage, and *ESM1* expression of each clone was determined by a quantitative RT-PCR for the selection of stably-transduced clone of MDA-MB-231/ESM1. The clone expressed *ESM1* close to that in MDA-MB-231BR was selected and named as MDA-MB-231/ESM1_D2. *ESM1* mRNA expression, endocan protein in the conditioned medium, and the proliferation rate of MDA-MB-231/ESM1_D2 were determined by a quantitative RT-PCR, ELISA, and a CCK-8 assay, respectively.

**Supplementary Fig. 1.** Kaplan-Meier survival curves of TNBC patients based on expressions of nine highest genes (other than *ESM1*) that are overexpressed in MDA-MB-231BR compared to MDA-MB-231.

**Supplementary Table 1.** PCR primers used in this study.

| Species |  | Accession  number |  | Primer sequences | Product size  (base pairs) |
| --- | --- | --- | --- | --- | --- |
| Human | RPS18 | NM_022551.2 | Forward | ATACAGCCAGGTCCTAGCCA | 96 |
|  |  |  | Reverse | AAGTGACGCAGCCCTCTATG |  |
|  | ESM1 | NM_007036.4 | Forward | TTGCTACCGCACAGTCTCAG | 126 |
|  |  |  | Reverse | GTGCCGTAGGGACAGTCTTT |  |
| Mouse | RPS18 | NM_011296.2 | Forward | ACTTTTGGGGCCTTCGTGTC | 102 |
|  |  |  | Reverse | GCAAAGGCCCAGAGACTCAT |  |
|  | ESM1 | NM_023612.3 | Forward | GCTACCGTACAGTCTCAGGC | 125 |
|  |  |  | Reverse | GGTGCCATAGGGACAGTCTTT |  |

**Supplementary Table 2.** Doubling time and ESM1 levels of TNBC cell lines.

| **A** | Doubling time ^†^  (hrs) | *ESM1* mRNA by qRT-PCR  (2^-ΔΔ^*^Ct^* values) | Endocan by ELISA  (ng/1 × 10^5^ cells) |
| --- | --- | --- | --- |
| MDA-MB-231SCP2 | 24.1 ^NS^ | 0.506 ^NS^ | 2.43 × 10^-2^ ^NS^ |
| MDA-MB-231/ESM1_D2 ^††^ | 21.5 ** | 1.61 × 10^3^ *** | 1.51 × 10^2^ *** |
| ^†^ Doubling times are expressed as median of six independent data, and compared with that of MDA-MB-231 (25.1 hr).  ^††^ Lentiviral human *ESM1* was transduced into MDA-MB-231, and several colonies were subcultured each passage. *ESM1* expression of each clone was determined by qRT-PCR. The clone expressed *ESM1* close to that in MDA-MB-231BR was selected and named as MDA-MB-231/ESM1_D2.  NS: No significance *vs.* MDA-MB-231; **: *P*<0.01 *vs.* MDA-MB-231; ***: *P*<0.001 *vs.* MDA-MB-231 | | | |
|  |  |  |  |

| **B** | Doubling time ^†^  (hrs) | *ESM1* mRNA by qRT-PCR  (2^-ΔΔ^*^Ct^* values) |
| --- | --- | --- |
| 4T1/luc2 | 15.8 | 1.63 × 10^-2^ |
| ^†^ Doubling time is expressed as median of six independent data. | | |
