# Supplementary figures and images for: Endocan as a prognostic biomarker of triple-negative breast cancer
Source: Breast Cancer Res Treat. 2016 Nov 25;161(2):269–78. doi: 10.1007/s10549-016-4057-8 (PMC5225208; doi:10.1007/s10549-016-4057-8)

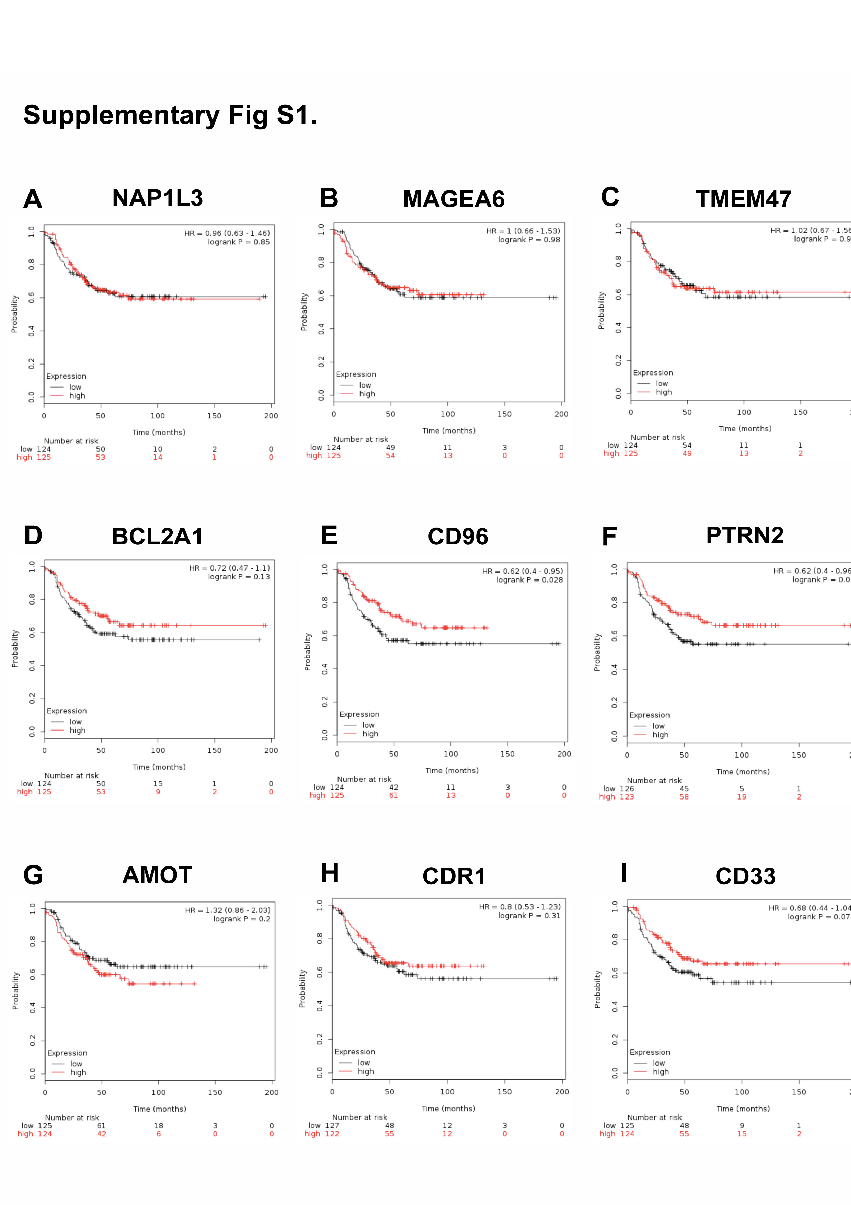

Supplement: Supplementary file 2 — Supplementary material 2 (DOCX 215 kb) [file 10549_2016_4057_MOESM2_ESM.docx]
